# Muscle-relevant genes marked by stable H3K4me2/3 profiles and enriched MyoD binding during myogenic differentiation

**DOI:** 10.1371/journal.pone.0179464

**Published:** 2017-06-13

**Authors:** Huanhuan Cui, Vikas Bansal, Marcel Grunert, Barbora Malecova, Alessandra Dall'Agnese, Lucia Latella, Sole Gatto, Tammy Ryan, Kerstin Schulz, Wei Chen, Cornelia Dorn, Pier Lorenzo Puri, Silke R. Sperling

**Affiliations:** 1 Department of Cardiovascular Genetics, Experimental and Clinical Research Center, Charité - Universitätsmedizin Berlin, Berlin, Germany; 2 Department of Biology, Chemistry, and Pharmacy, Freie Universität Berlin, Berlin, Germany; 3 Berlin Institute of Health (BIH), Berlin, Germany; 4 Department of Mathematics and Computer Science, Freie Universität Berlin, Berlin, Germany; 5 Development Aging & Regeneration Program, Sanford-Burnham-Prebys Medical Discovery Institute, La Jolla, California, United States of America; 6 Epigenetics and Regenerative Medicine, Istituto di Ricovero e Cura a Carattere Scientifico Fondazione Santa Lucia, Rome, Italy; 7 Max Delbrück Center for Molecular Medicine in the Helmholtz Association, Berlin Institute for Medical Systems Biology, Berlin, Germany; Università degli Studi di Milano, ITALY

## Abstract

Post-translational modifications of histones play a key role in the regulation of gene expression during development and differentiation. Numerous studies have shown the dynamics of combinatorial regulation by transcription factors and histone modifications, in the sense that different combinations lead to distinct expression outcomes. Here, we investigated gene regulation by stable enrichment patterns of histone marks H3K4me2 and H3K4me3 in combination with the chromatin binding of the muscle tissue-specific transcription factor MyoD during myogenic differentiation of C2C12 cells. Using *k*-means clustering, we found that specific combinations of H3K4me2/3 profiles over and towards the gene body impact on gene expression and marks a subset of genes important for muscle development and differentiation. By further analysis, we found that the muscle key regulator MyoD was significantly enriched on this subset of genes and played a repressive role during myogenic differentiation. Among these genes, we identified the pluripotency gene *Patz1*, which is repressed during myogenic differentiation through direct binding of MyoD to promoter elements. These results point to the importance of integrating histone modifications and MyoD chromatin binding for coordinated gene activation and repression during myogenic differentiation.

## Introduction

Myogenic differentiation is an essential process of muscle development and depends on the spatiotemporal regulation of gene expression patterns. Understanding the molecular and epigenetic regulation of gene expression during myogenic differentiation is important to gain insights into the mechanism underlying the pathogenesis of muscular dystrophies [1,2]. Epigenetic marks such as histone modifications play an important role in the regulation of gene transcription and different histone marks are related to distinct regulatory elements [3]. These modifications can recruit further transcriptional regulators and consequently modulate the expression of *cis*-regulated genes [4]. For example, di- and tri-methylation of lysine 4 on histone 3 (H3K4me2 and H3K4me3, respectively) are generally associated with euchromatin and active gene expression [5]. In human hematopoietic cells, both modifications are mostly found at transcribed promoters and show distinct profiles with H3K4me3 located towards the gene body and H3K4me2 extending into the gene body [6,7]. Moreover, a recent study revealed that a subset of tissue-specific genes was characterized by H3K4me2 within the gene body in human CD4+ T cells and neural tissue [8]. In general, the lysine methylation is generated by histone methyltransferases and reversed byhistone demethylases, which play an important role in myogenic differentiation. For instance, the SET domain-containing methyltransferases MLL5 and SET7 regulates myogenic differentiation by controlling cell cycle genes and myogentic regulator genes [9,10]. During myogenic differentiation, the overall content of histone methylations such as H3K4me2, H3K4me3, H3K36me3 and H3K27me3 were shown to be stable [11]. The repressive histone mark H3K27me3 was found to be widely distributed throughout the genome and regulates myogenic differentiation via silencing of muscle-specific and cell cycle genes [11–13]. However, histone 3 acetylations like H3K9ac and H3K18ac are reduced in a differentiation-dependent manner [11]. Furthermore, different histone marks and transcription factors (TFs) form a combinatorial code, which leads to distinct outcomes and modulates gene expression [14,15]. Interestingly, the regions of increased histone 4 acetylation (H4Kac) have been associated with the genome-wide binding of the master myogenic transcription factor MyoD (MyoD1; myogenic differentiation 1) [16,17].

MyoD belongs to the myogenic basic helix-loop-helix (bHLH) family of transcription factors that activate muscle-gene expression during skeletal myogenesis through DNA binding to the consensus E-box motif (CANNTG) [18]. Upon the induction of differentiation, MyoD forms heterodimers with members of the E-protein family with an increased affinity at many regulatory elements of skeletal muscle-specific genes [19,20]. In undifferentiated myoblasts, MyoD and Baf60C, a subunit of the ATPase-containing SWI/SNF remodeling complex, form a complex on MyoD target promoters and mark genes prior to the activation of transcription, which play a role in myogenic differentiation [21]. During myogenesis, the binding of MyoD is primarily associated with gene activation [22], but its repressive function in myogenesis has also been shown on single genes [23–25]. In addition to promoters, genome-wide analysis has indicated MyoD binding events in intergenic regions in myoblasts and myotubes [16,26]. Moreover, the presence of MyoD is also highly associated with muscle-related enhancers [27].

Here, we investigated a stable enrichment pattern of the histone marks H3K4me2 and H3K4me3 in combinations with muscle tissue-specific transcription factor MyoD during myogenic differentiation. We performed chromatin immunoprecipitation followed by next-generation sequencing (ChIP-seq) in two stages of myogenic differentiation in C2C12 cells. To study the impact on gene expression, we further analyzed genome-wide expression data (RNA-seq) from the ENCODE project [28]. Finally, we extended and validated our results using skeletal muscle cells generated from fibroblasts. In summary, we could identify a subset of highly expressed muscle-related genes, which show stable and distinct H3K4me2 and H3K4me3 profiles and an enrichment of MyoD binding. Moreover, we show that during myogenic differentiation MyoD plays a repressive role on this subset of H3Kme2/3 marked genes, which include the pluripotency transcription factor Patz1.

## Materials and methods

### Cell culture

C2C12 myoblasts were obtained from Professor Jakob Schmidt (Department of Biochemistry and Cell Biology, State University of New York, Stony Brook, NY, USA) and cultured at 5% CO_2_ and 37°C in Dulbecco's modified Eagle's medium (DMEM; Gibco) supplemented with 1% penicillin/streptomycin (Gibco) and 10% fetal bovine serum (Biochrom). Mononucleated C2C12 myocyte cells were harvested before reaching 70% confluence. To induce differentiation, cells were cultured with Dulbecco's modified Eagle's medium and 2% horse serum (Biochrom) and maintained for 48 hours, when more than 90% of the cells had fused into myotubes.

IMR-90 and BJ-CRL2522 primary human fibroblasts were purchased from ATCC. IMR-90 fibroblasts were cultured in growth medium supplemented with 10% fetal bovine serum. Muscle differentiation was induced by incubating cells in DMEM supplemented with 2% horse serum, 0.01 mg/ml bovine insulin and 0.0055 mg/ml human transferrin. BJ fibroblasts were cultured in growth medium supplemented with 10% fetal bovine serum, non-essential amino acids, pyruvate and penicillin–streptomycin. The differentiation was induced by incubating cells in DMEM supplemented with 2% horse serum.

HEK293 cells (ATCC) were cultured at 5% CO_2_ and 37°C in Dulbecco's modified Eagle's medium (DMEM; Gibco) supplemented with 1% penicillin/streptomycin (Gibco) and 10% fetal bovine serum (Biochrom).

### Transfection of human fibroblasts

IMR-90 fibroblasts were transfected with a MyoD expression construct by electroporation. Cells were selected with 2 mg/ml of puromycin for six nights. MyoD expression was induced by 200 ng/ml of doxycycline in growth medium for 24 hours. No doxycycline was added in control cells. Differentiation was induced by culturing cells in differentiation medium for three days with/without doxycycline for 72 hours.

To transfect BJ fibroblasts, MyoD ectopic expression was achieved by Adenoviral infection with Adeno-MyoD at MOI 800 for one hour. Adeno-Track was used as control virus. Then, cells were placed in growth medium for 24 hours and differentiation was induced by changing cells to differentiation medium for 56 hours. MyoD expression levels were measured by real-time PCR 24 hours after infection.

### Antibodies, ChIP, and ChIP-seq

ChIP was performed as described before [29]. Briefly, the MAGnify^™^ Chromatin Immunoprecipitation System (Life Technologies, 49–2024) was used with some modifications. Sonication was performed using the Biorupter UCD300 (Diagenode) to obtain chromatin fragments of approximately 100–300 bp. The following antibodies were used for ChIP: anti-H3K4me2 (Abcam ab7766), anti-H3K4me3 (Abcam ab8580), and anti-MyoD (Santa Cruz, sc-760). Sequencing libraries were prepared using the NEXTflex^™^ ChIP-Seq Kit (Bio Scientific, 5143) according to an in-house modified protocol. The libraries were 51 bp single-end sequenced on an Illumina HiSeq 2000 platform. Base calling was performed with the Illumina Casava pipeline version 1.8.0. Initial sequencing quality assessment was based on data passing the Illumina Chastity filter. The raw and processed ChIP-seq data was submitted to GEO (GSE63716).

### RNA extraction and real-time PCR

Total RNA from C2C12, IMR-90 and BJ cells was extracted with Trizol (Invitrogen), according to manufacturer’s instructions. 0.5–1 μg of RNA was retro-transcribed using the Taqman reverse transcription kit (Applied Biosystems). Real-time quantitative PCR was performed using a 7500 Fast Real-Time System machine to analyze relative gene expression levels using SYBR Green Master mix (Applied Biosystems) and following manufacturer’s indications. Gene expression was calculated using the delta-CT method with normalization to the housekeeping gene *Hprt*. For ChIP samples, enrichments were measured relative to input.

### Reporter gene assay

A minimum 400 bp *Patz1* promoter (chr11:3190209–3190608, mm9) was cloned into the pGL3 basic vector (Promega). For luciferase assays, approximately 10^4^ HEK293 cells were transiently transfected with 50ng of reporter vector, 5 ng of Firefly luciferase vector for internal normalization of transfection efficiency and 10–200 ng of MyoD expression vectors using Transfast (Promega). Activity was measured by Dual-Luciferase assay (Promega) after 48 hours in a Centro LB960 Luminometer (Berthold). All measurements were performed in triplicates.

### ChIP-seq analysis

Sequencing of DNA libraries resulted in ~29–66 million reads per sample (S1 Table). Quality assessment was based on the raw reads using the FASTQC quality control tool (v0.10.1) [30]. The sequence reads (single-end 51 bp) were mapped to the mouse reference genome (mm9) using Bowtie (v0.12.9) [31] with default parameters. Ambiguously mapped reads were discarded from further analysis. Replicate BAM files were merged using SAMtools (v0.1.18.0) [32].

For signal detection (H3K4me2/3) and peak calling (MyoD), we used MACS (v1.4.2) [33], which provides peaks and fitted signals. The shift size parameter was set according to the obtained fragment sizes (S1 Table). For peak detection a *P* value cutoff of 10^−4^ was used.

To assign MyoD peaks to genes and histone signals to TSS, RefSeq genes (mm9) from the UCSC genome browser were used, containing 23,358 genes corresponding to 30,089 transcripts. MyoD peaks were assigned to the genes if they are located within 10 kb upstream of the TSS or in the transcribed region. For the analysis of histone signals, we focused on unique transcripts with at least 4 kb in length. The filtering resulted in 19,904 TSS corresponding to 24,051 transcripts (S1 Fig). For each TSS, ChIP-seq signal were assigned 2 kb upstream and 4 kb downstream. The resulting region of 6 kb length was further divided in 100 bp long non-overlapping windows and the total signal was calculated for each of these windows. Furthermore, signals were normalized (per million reads).

### K-means clustering

Before clustering, the input signal was subtracted from the H3K4me2/3 signal for each TSS region (6 kb) and regions with signal ≤ 0 were discarded (S1 Fig). *K*-means clustering was performed in R (v3.0.2) using the “kmeans” function with centers = 6 and iter.max = 1000000. We tried centers = 4,5,6,7 clusters and manually visualize if the clusters can be further separated with increasing centers. We defined six clusters for histone marks based on the visual inspection.

### Gene ontology enrichment analysis

GO analysis was conducted using the DAVID functional annotation tool [34]. An adjusted *P* value ≤ 0.01 using the Benjamini-Hochberg method for controlling the false discovery rate was set as significant for GO terms in biological processes. To validate the results, we used 324 genes related to “muscle organ development” and 406 genes related to “muscle tissue development” based on the Mouse Genome Informatics (MGI) database [35]. Using the two-sided Fisher's exact test, we confirmed that genes in Undiff H3K4me2 cluster 1, Diff H3K4me2 cluster1, Undiff H3K4me3 cluster 1 and Diff H3K4me3 cluster 1 are significantly enriched for both terms (*P* values < 10^−7^).

### RNA-seq analysis

RNA-seq data was used from the ENCODE project [28]. The 75 bp long reads (204 and 185 million paired-end reads in undifferentiated and differentiated C2C12 cells, respectively) were mapped to the mouse reference genome (mm9) using TopHat (v2.0.8) [36] with default parameters. FPKM values were calculated using the Cufflinks (v2.0.2) [37] with default parameters.

### Statistics

General bioinformatics and statistical analyses were conducted using R (including Bioconductor packages) and Perl.

### Data access

ChIP-seq data are available from the Gene Expression Omnibus (GEO) repository at NCBI (accession number GSE63716). RNA-seq data in C2C12 cells from ENCODE [28] are available from the Sequence Read Archive (SRA) at NCBI with accession numbers SRR496442 (undifferentiated C2C12 cells) and SRR496443 (differentiated C2C12 cells).

## Results

### Study design

C2C12 mouse myoblasts [38] provide the most used experimental model to investigate epigenetic profiles that underlie myogenic differentiation (9, 14, 19, 23, 24). We performed ChIP-seq for H3K4me2, H3K4me3 and MyoD in C2C12 cells cultured either in growth medium (GM)—undifferentiated myoblasts (Undiff)—or after exposure to differentiation medium (DM) for 48 hours—differentiated myotubes (Diff). In addition, expression profiles of Undiff and Diff C2C12 cells were obtained by RNA-seq from ENCODE [28]. Moreover, we analyzed myoblasts and myotubes that were generated from human fibroblasts by induction of MyoD expression. Fibroblasts were obtained from lung tissue (IMR-90) and normal foreskin (BJ-CRL2522), respectively [39,40]. In both myoblasts and myotubes generated from IMR-90 cells, ChIP-seq was performed for MyoD and in addition, gene expression was analyzed in both stages by RNA-seq.

### H3K4me2 over the gene body of muscle-specific genes

To analyze the distribution of histone modifications around transcriptional start sites (TSS), we filtered for a defined set of gene transcripts longer than 4 kb, resulting in 24,051 transcripts with 19,904 unique TSS (S1 Fig). We further analyzed regions from -2 kb to +4 kb around TSS, which enables the direct comparison of epigenetic profiles independent from the gene length [8]. For each C2C12 ChIP-seq sample, the transcripts with a lower H3K4me2 signal around the TSS as compared to the input sample were discarded, which resulted in approximately 18,000 to 20,000 transcripts per sample (S1 Fig). For each TSS, we generated the average ChIP-seq profile based on the normalized signal.

**Fig 1:**
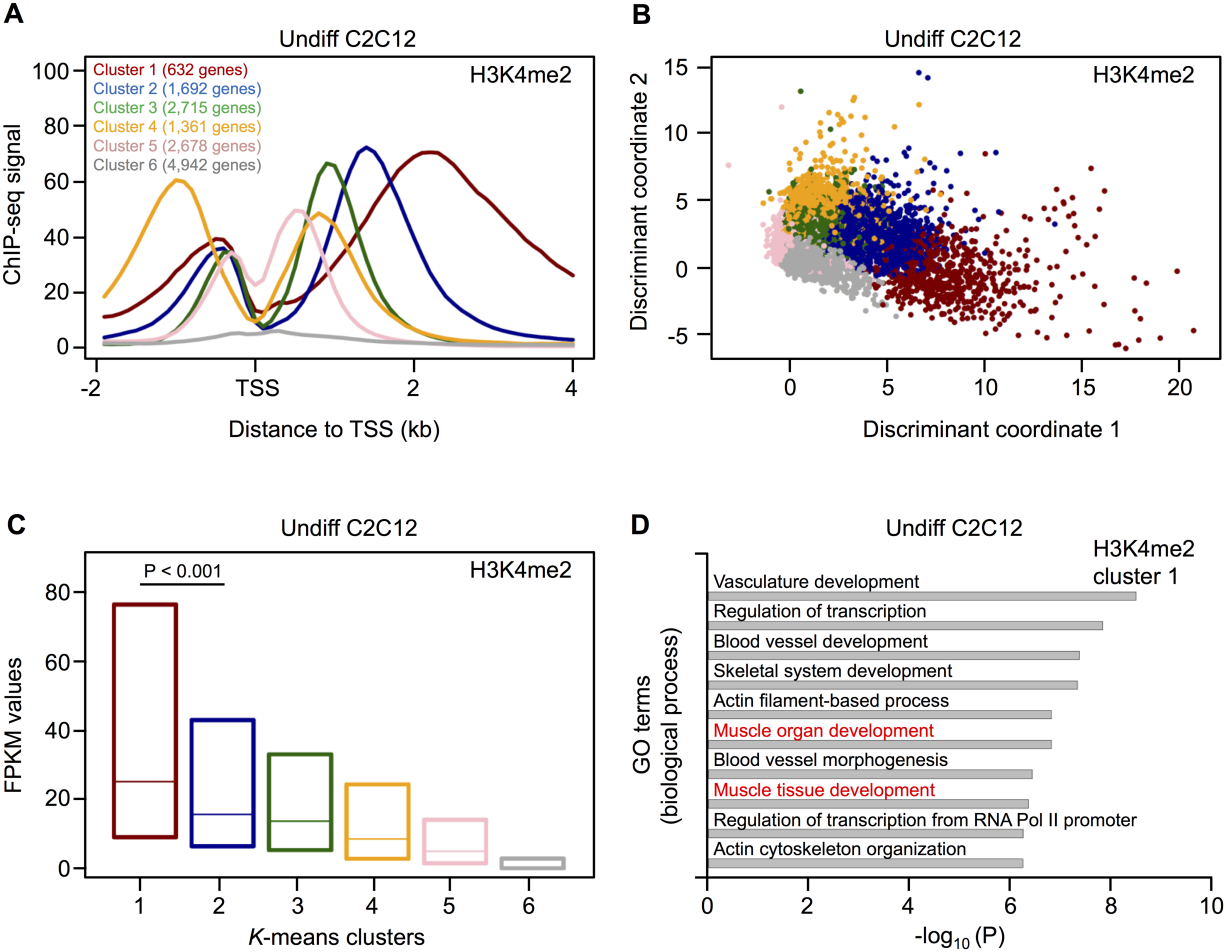
Clustering analysis of H3K4me2 profiles in undifferentiated C2C12 cells. (A) H3K4me2 profiles identified by *k*-means clustering. The clustering is based on TSS and the corresponding number of genes is given for each cluster. Genes with multiple TSS can be present in more than one cluster. (B) Discriminant analysis shows a clear distinction of the six clusters identified by *k*-means clustering. (C) The box plot (25% to 75% quartile) shows the levels of gene expression from the different H3K4me2 clusters in Undiff C2C12 cells. The expression of cluster 1 and cluster 2 genes was compared using the Mann-Whitney U test. (D) GO enrichment analysis of cluster 1 genes using the DAVID functional annotation tool. Top ten biological process terms with an adjusted (Benjamini-Hochberg) *P* value ≤ 0.01 are indicated. GO terms related to muscle development are highlighted in red.

In undifferentiated C2C12 cells, the average profile of H3K4me2 showed a bimodal distribution and revealed the highest enrichment downstream of the TSS (S2 Fig). To check whether any specific set of genes show distinct enrichment within the gene body, we performed *k*-means clustering with six clusters using the filtered set of TSS (Fig 1A). The first five clusters are characterized by specific distributions of H3K4me2 around the TSS. Here, cluster 1 (representing 632 genes) and cluster 4 (representing 1,361 genes) are the most distinct groups. Cluster 1 is characterized by H3K4me2 positioned over the gene body, while cluster 4 shows a higher prevalence upstream of the TSS. In contrast, cluster 6 includes genes with a very low H3K4me2 signal from -2kb to 4kb around the TSS (4,942 genes, Fig 1A). Finally, we verified the results of *k*-means clustering by discriminant analysis and found that the observed clusters can indeed be clearly distinguished from each other (Fig 1B).

When we combined the ChIP-seq data with gene expression profiles obtained by RNA-seq from ENCODE [28], we found that genes located in cluster 1 are significantly higher expressed compared to all other clusters (*P* value < 0.001). The lowest expression was found for genes detected in cluster 6 (Fig 1C). Further, we performed a GO enrichment analysis within each cluster using the DAVID functional annotation tool [34]. In contrast to all other clusters, cluster 1 genes are significantly enriched for GO terms related to muscle development (Fig 1D and S2 Table).

Next, we performed the same analysis of H3K4me2 profiles and related gene expression for differentiated C2C12 cells and obtained results comparable to the undifferentiated cells (S2 and S3 Figs). Again, significant GO terms related to muscle development were observed for cluster 1 genes (S3 Table). Then, we compared the clusters between undifferentiated and differentiated C2C12 cells and found that a high proportion of genes remained in the same cluster after differentiation (S3 Fig). For example, 83% of cluster 1 genes show a stable profile, with the same H3K4me2 distribution in both undifferentiated and differentiated C2C12 cells. Most interestingly, GO analysis of the stable (overlap between Undiff and Diff) and dynamic (specific for Undiff or Diff) gene sets in cluster 1 revealed that only the stable gene set is significantly enriched for muscle-related GO terms (S4 Table).

### H3K4me3 towards the gene body of muscle-specific genes

**Fig 2:**
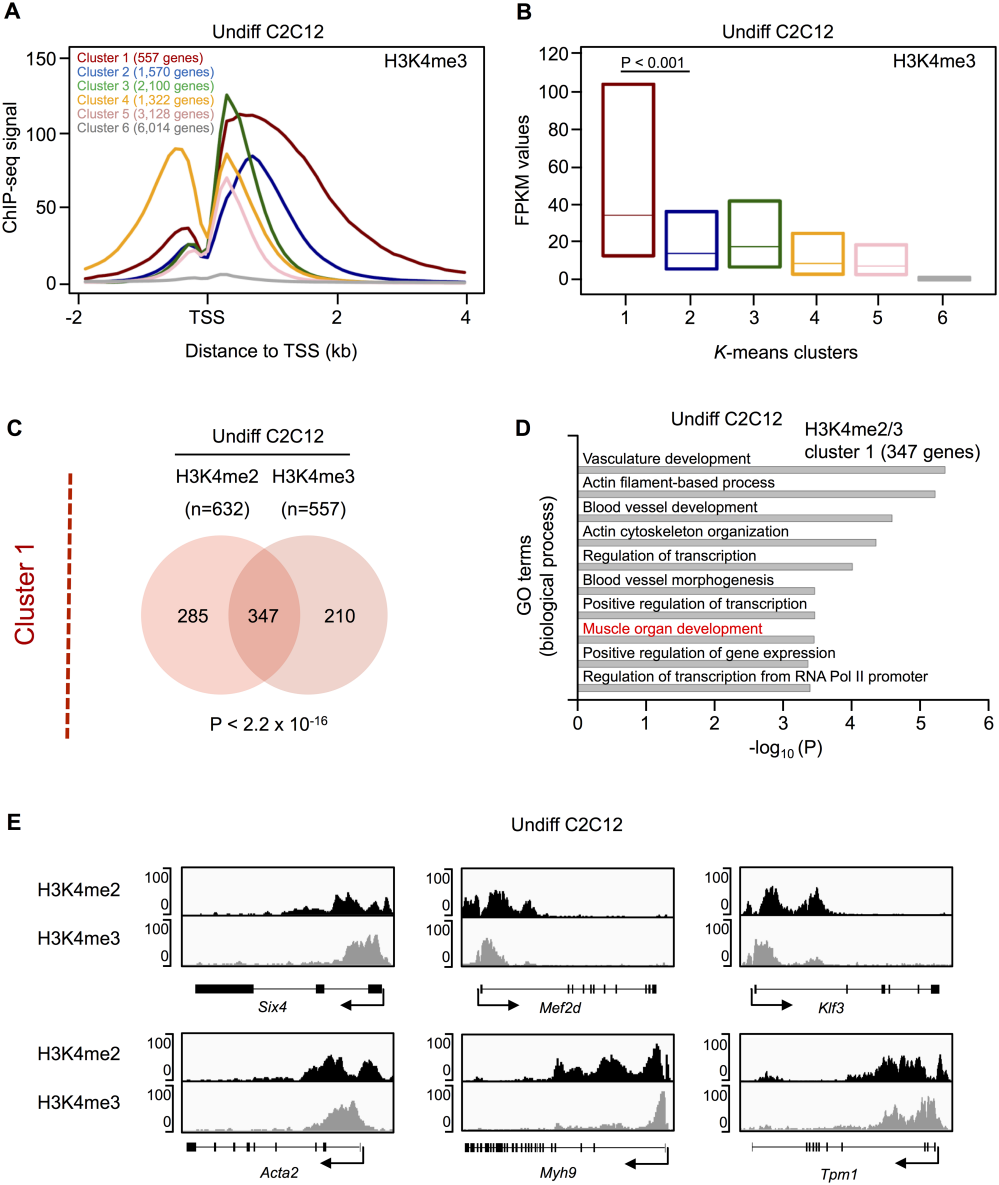
Clustering analysis of H3K4me3 profile in undifferentiated C2C12 cells and comparison to H3K4me2 profiles. (A) H3K4me3 profiles identified by *k*-means clustering. The clustering is based on TSS and the corresponding number of genes is given for each cluster. Genes with multiple TSS can be present in more than one cluster. (B) The box plot (25% to 75% quartile) shows the levels of gene expression from the different H3K4me3 clusters in Undiff C2C12 cells. The expression of cluster 1 and cluster 2 genes was compared using the Mann-Whitney U test. (C) Overlap of H3K4me2 and H3K4me3 cluster 1 genes in Undiff C2C12 cells. The *P* value is based on a hypergeometric test. (D) GO enrichment analysis of common cluster 1 genes using the DAVID functional annotation tool. Top ten biological process terms with an adjusted (Benjamini-Hochberg) *P* value ≤ 0.01 are indicated. GO terms related to muscle development are highlighted in red. (E) H3K4me2 and H3K4me3 enrichment profiles of selected muscle-relevant cluster 1 genes. The TSS is marked by an arrow. The y-axis indicates the ChIP-seq signal.

Compared to H3K4me2, we observed a higher mean enrichment of H3K4me3 directly downstream of the TSS in both undifferentiated and differentiated C2C12 cells (S2 Fig). Moreover, clustering identified distinct profiles for both myoblasts and myotubes, with cluster 1 genes showing H3K4me3 enrichment towards the gene body (Fig 2A and S4 Fig). In addition, cluster 1 genes are significantly higher expressed as compared to the remaining clusters (*P* value < 0.001, Fig 2B and S4 Fig) and show a significant enrichment of GO terms related to muscle development (S5 and S6 Tables). When we compared the H3K4me3 clusters between Undiff and Diff C2C12 cultures, we found that a high proportion of genes remained in the same cluster. However, H3K4me3 profiles are more dynamically distributed among genes, with higher number of genes changing their clusters during differentiation (S4 Fig). For example, only 71% of cluster 1 genes have a stable H3K4me3 profile, while 83% of cluster 1 genes have a stable H3K4me2 profile. As for H3K4me2, genes in cluster 1 with a stable H3K4me3 profile are significantly enriched for GO terms related to muscle development (S7 Table). Finally, the GO terms “muscle organ development” and “muscle tissue development” were further confirmed for cluster 1 in H3K4me2 as well as H3K4me3 in undifferentiated and differentiated C2C12 cells (*P* values < 10^−7^) using the MGI database [35].

Given that muscle-related genes (based on GO analysis) are enriched in cluster 1 of H3K4me2 and H3K4me3, we analyzed the overlap between these clusters in undifferentiated C2C12 cells. We found a significant overlap of genes (*P* value < 2.2x10^-16^, Fig 2C) and a significant enrichment of GO terms related to muscle development for these 347 common cluster 1 genes (Fig 2D). Comparable results were obtained in differentiated C2C12 cells (S5 Fig).

Fig 2E and S6 Fig shows the H3K4me2/3 profiles and ChIP-qPCR validation for a subset of common cluster 1 genes, which plays an important role in muscle cell development, differentiation and regeneration. These examples illustrate the different distributions of these two histone marks, with H3K4me2 located over and H3K4me3 towards the gene body. Among these genes, the transcription factor Six4 (SIX Homeobox 4) directly activates MyoD expression in gene regulatory networks that control early myogenesis [41,42]. Mef2d (Myocyte Enhancer Factor 2D) is an early marker of the myogenic lineage and is required for skeletal muscle regeneration [43,44]. Klf3 (Kruppel-Like Factor 3) synergizes with serum response factor on KLF binding sites to regulate muscle-specific gene expression [45]. The myogenic factor Tpm1 (Tropomyosin 1) is essential for myotube formation and plays a pivotal role in regulating muscle contraction [46]. Acta2 (Actin, Alpha 2, Smooth Muscle, Aorta) and Myh9 (Myosin, Heavy Chain 9, Non-Muscle) belong to the actin and the myosin family of proteins, respectively, which are essential for muscle cell structure and mobility [47,48].

### Integrating H3K4 methylation with gene expression and MyoD binding profile

**Fig 3:**
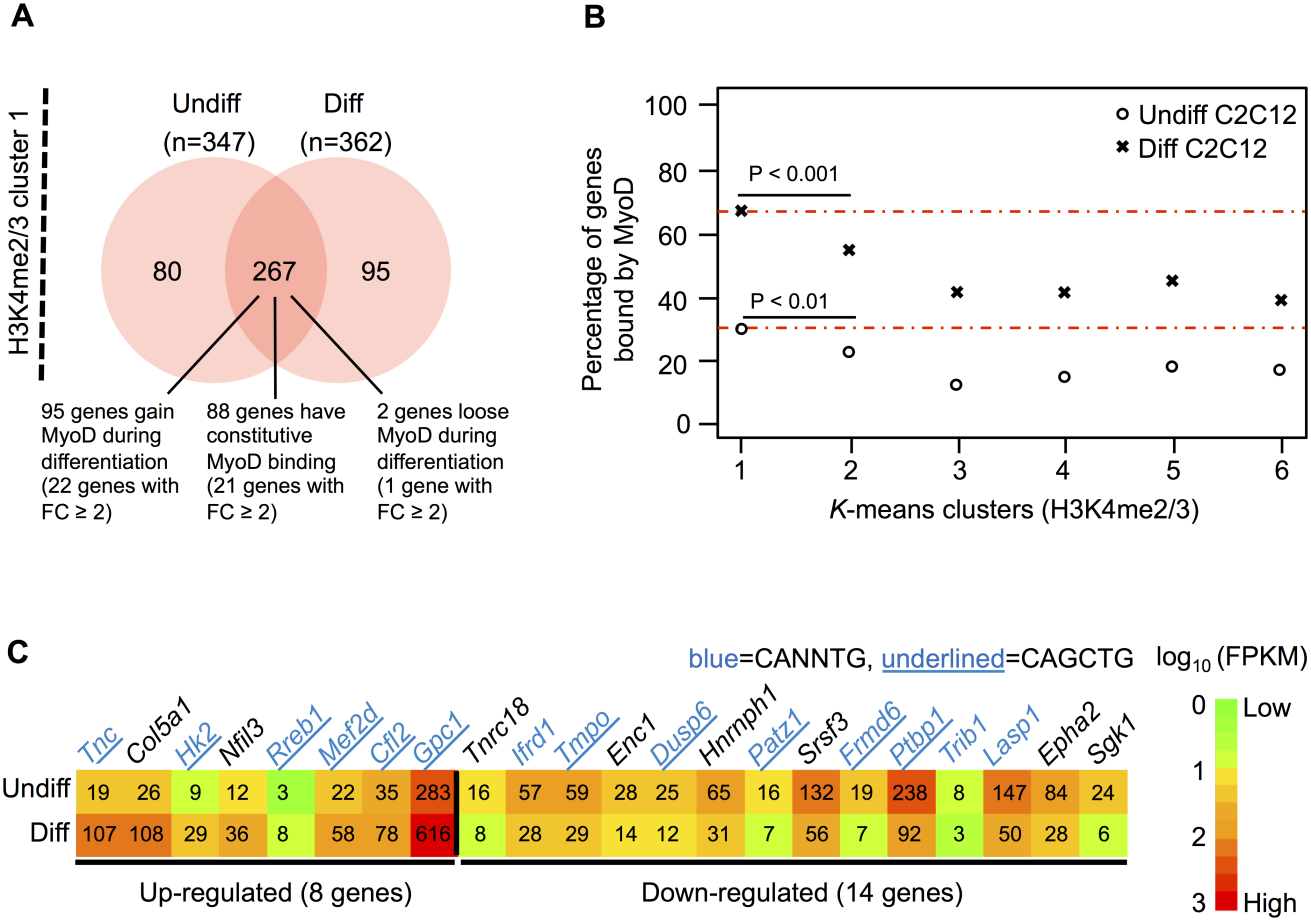
Cluster 1 genes bound by MyoD. (A) Overlap of common H3K4me2/3 cluster 1 genes in Undiff and Diff C2C12 cells. The number of genes, which gain, loose or have constitutive MyoD binding are indicated, including their respective number of differentially expressed genes (fold change (FC) ≥ 2). (B) Percentage of genes in the common H3K4me2/3 clusters bound by MyoD in Undiff and Diff C2C12 cells. Highest enrichment in Undiff and Diff (each in cluster 1) is indicated by the two red lines. The *P* values are based on two-sided Fisher's exact test. (C) Heatmap of differentially expressed genes in H3K4me2/3 cluster1, which gain MyoD during differentiation (22 genes with fold change ≥ 2 out of 95 genes). The numbers in the heatmap represent the FPKM (fragments per kilo bases of exons for per million mapped) values. Gene names in blue indicate the genes with the E-box motif (CANNTG) within a 30 bp region centered on the peak summit. Gene names underlined in blue are genes with the MyoD preferred E-box motif (CAGCTG) within a 30 bp region centered on the peak summit.

To identify muscle-relevant genes with a stable H3K4 di- and tri-methylation profile, we overlapped the 347 common cluster 1 genes from undifferentiated C2C12 cells with the 362 common cluster 1 genes from the differentiated stage. This resulted in a total of 267 genes with stable H3K4me2 and H3K4me3 profiles over or towards the gene body, respectively (Fig 3A). Of note, these genes were devoid of the PCR2-mediated repressive mark H3K27me3 (data not shown).

As expected, these 267 genes are significantly enriched for GO terms related to muscle development (S8 Table). We then investigated the binding profile of MyoD on common stable cluster 1 genes, by performing ChIP-seq analysis MyoD genome-wide binding in Undiff and Diff C2C12 cells. For ChIP-seq data, we performed peak calling using MACS [33] and assigned peaks to the genes if they are located within 10 kb upstream of the TSS or in the transcribed region. Out of our filtered set of genes (S1 Fig), 15% (2,618 genes) were bound by MyoD in undifferentiated C2C12 cells, while 38% (6,774 genes) show a MyoD peak in differentiated myotubes. This is in line with previous studies showing an enrichment of MyoD binding during differentiation [16,49].

The percentage of genes bound by MyoD for each common H3K4me2/3 cluster is given in Fig 3B for both differentiation stages. Interestingly, cluster 1 harbors a significantly higher percentage of genes bound by MyoD compared to all other clusters (*P* value < 0.01 in Undiff and *P* value < 0.001 in Diff). In this cluster, we found approximately 30% of genes bound by MyoD in Undiff and 67% in Diff C2C12 cells (S9 Table). Focusing again on the common stable cluster 1, out of the 267 genes, 95 of them show a further enrichment in MyoD binding during differentiation, with 23% (22 genes) being differentially expressed (Fig 3A). Given that MyoD is a transcriptional activator [50,51], we were expecting most of these genes to be up-regulated. Interestingly, we found 64% of this specific set of genes (14 out of 22) to be down-regulated (Fig 3C). The presence of MyoD on promoters of both up- and down-regulated genes suggests that MyoD can either activate or repress the expression of genes marked by H3K4me2/3. To investigate this issue, we further searched for the MyoD binding E-box motif (CANNTG) within a 30bp region centered on the peak summit in these 22 differentially expressed genes. We found 14 genes harboring this motif (Fig 3C), of which 11 contain a particular E-box motif (CAGCTG) in which the central nucleotide has been shown to confer preferential affinity for MyoD [16]. This “private” E-box motif discriminates the ability to activate muscle gene expression by MyoD from that of the neurogenic bHLH NeuroD to activate the expression of neuronal genes. Interestingly, the same E-box motif was found under the MyoD peaks identified in five down-regulated genes (Fig 3C), including *Dusp6* (dual specificity phosphatase 6), *Frmd6* (FERM domain containing 6), *Patz1* (POZ (BTB) and AT hook containing zinc finger 1), *Ptbp1* (polypyrimidine tract binding protein 1) and *Tmpo* (thymopoietin).

To further investigate the repression role of MyoD on these 14 down regulated genes during myogenic differentiation, IMR-90 human fibroblasts were converted to skeletal muscle cells by induction of MYOD. The expression of MYOD was induced ~400-fold in growth medium (GM) and ~800-fold in the differentiation medium (DM) (S7 Fig). Interestingly, 9 and 10 genes out of these 14 genes were indeed down regulated by the induction MYOD in GM and DM, respectively (S10 Table), including the zinc finger transcription factor *Patz1*, which was previously shown to have an important role in maintenance of the embryonic stem cell (ESC) phenotype and its knockdown leads to differentiation of murine ESCs into endoderm and mesoderm lineages at different time points [52].

### Down-regulation of Patz1 by MyoD during myogenic differentiation

**Fig 4:**
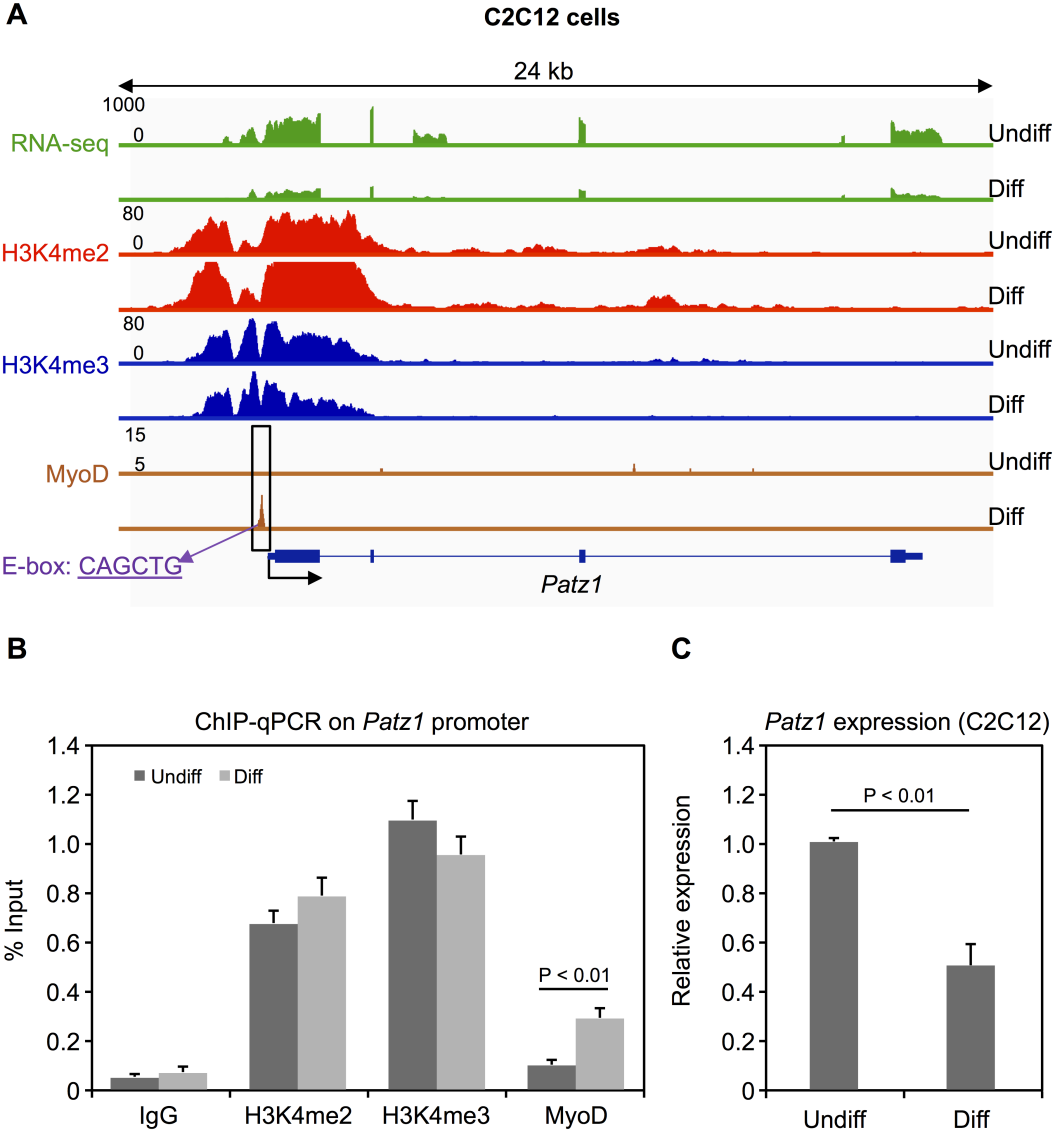
*Patz1* expression and MyoD binding during myogenic differentiation. (A) RNA expression profile of *Patz1*, H3K4me2 enrichment profile, H3K4me3 enrichment profile and MyoD binding profile at the *Patz1* promoter in Undiff and Diff C2C12 cells. All profiles are based on raw mapped reads. Position of the MyoD preferred E-box motif (CAGCTG) in the peak region is indicated. (B) ChIP analysis of MyoD occupancy levels and H3K4me2/3 enrichment at the Patz1 promoter. Error bars indicate the standard deviation from at least three independent experiments. The statistical significance of enrichment versus the IgG control was calculated using Student's t-test. (C) Expression levels (mRNA) of Patz1 in Undiff and Diff C2C12 cells were measured by real-time PCR in at least three independent experiments. The statistical significance of the difference in expression between Undiff and Diff C2C12 was calculated using Student's t-test.

We further investigated *Patz1* expression pattern, as a representative gene among those exhibiting H3K4me2/3 profile and bound by MyoD only in Diff C2C12 cells (Fig 4A). *Patz1* expression and MyoD binding to *Patz1* promoter were monitored by real time PCR and ChIP-qPCR during C2C12 differentiation, confirming the down-regulation and promoter occupancy by MyoD during myotube formation (Fig 4B and 4C, respectively). These results indicate that the binding of MyoD can also correlate with gene down-regulation of target genes during differentiation of C2C12 cells.

**Fig 5:**
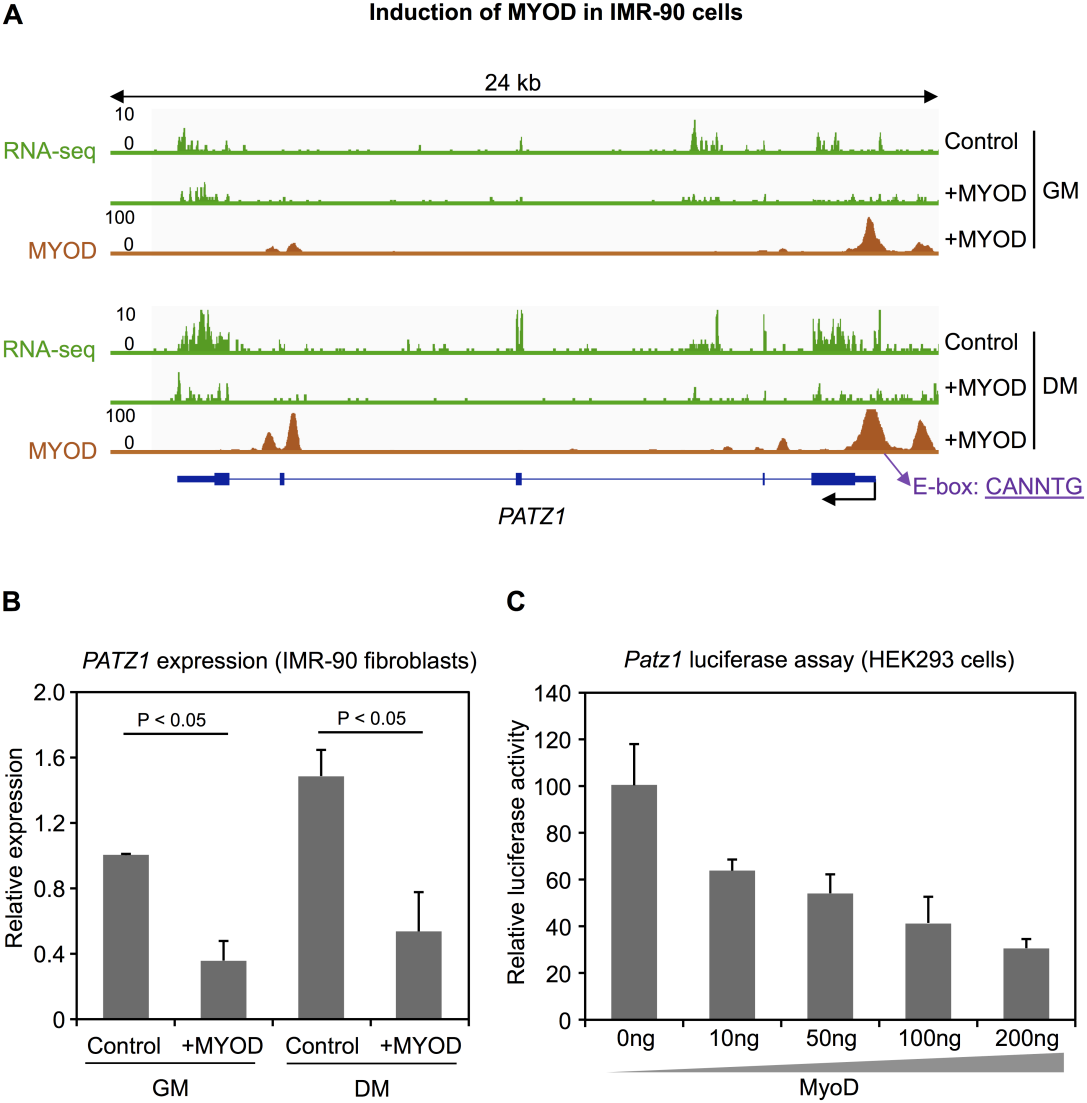
*PATZ1* down-regulation by MyoD. (A) RNA expression profile of *PATZ1* and MYOD binding profile at the *PATZ1* promoter in IMR-90 cells cultured with growth medium (GM) and differentiation medium (DM). All profiles are based on raw mapped reads. Position of the MyoD E-box motif (CANNTG) in the peak region is indicated. (B) Expression levels (mRNA) of *PATZ1* in IMR-90 fibroblasts. *P* value was calculated using Student's t-test based on at least three independent experiments. (C) Luciferase reporter analysis in HEK293 cells transiently transfected with a *Patz1* reporter-construct and MyoD expression plasmids or empty vector control. Measurements were performed in triplicates.

Based on the ChIP-seq data in IMR-90 human fibroblasts, we found MYOD binding at the *PATZ1* promoter in both induced stages of converting fibroblasts to skeletal muscle cells (Fig 5A). Moreover, MYOD binding in the DM stage was higher compared to GM treated IMR-90 cells. Upon induction of MYOD, the expression of *PATZ1* was significantly down-regulated in both stages (Fig 5A and 5B). The same data were obtained by ectopic MYOD expression in another type of human primary fibroblasts (BJ fibroblasts) (S7 Fig).

Finally, we performed luciferase reporter gene assays to study the role of the binding of MyoD at the *Patz1* promoter *in vitro*. Assays were performed by co-transfection of a MyoD expression vector together with a *Patz1* promoter reporter vector in HEK293 cells. The 400 bp core promoter of *Patz1* was efficiently repressed by co-transfection of MyoD in a dosage dependent manner (Fig 5C).

Taken together, these findings suggest that MyoD binds to the *Patz1* promoter and down-regulates *Patz1* expression to promote myogenic differentiation. Our results underline that in addition to activating muscle-specific genes, MyoD also acts as a repressor during myogenesis.

## Discussion

In this study, we analyzed H3K4me2/3 signatures in myogenic differentiation and found specific profiles on muscle-relevant genes. In general, the average profile of H3K4me3 is enriched directly downstream of the TSS, whereas H3K4me2 is further located over the gene body, as previously shown also in hematopoietic cells [6,7]. To identify specific H3K4me2/3 profiles, we used *k*-means clustering to define six groups of genes, showing distinct H3K4me2 and H3K4me3 patterns, respectively. *K*-means clustering has been shown to be useful in partitioning the distinct enrichment patterns of histone modifications [8,53]. We identified one cluster (cluster 1) with a H3K4 methylation profile over the gene body (di-methylation—H3K4me2) or towards the gene body (tri-methylation—H3K4me3), respectively. Cluster 1 genes are significantly higher expressed than all other clusters and moreover, are significantly enriched for GO terms related to muscle development. This is in line with a previous study, showing a similar H3K4me2 profile of tissue-specific genes in CD4+ cells and brain tissue [8]. In addition, we could show that a unique profile (cluster 1) of H3K4me3 also marks muscle-specific genes.

Next, we matched the clusters identified in undifferentiated and differentiated C2C12 cells and found a high proportion of genes remaining in the same cluster for both methylation profiles, with H3K4me2 profiles being even more stable than H3K4me3 profiles. For acetylation of H3K9, H3K18 and H4K12, a striking reduction have been previously described during myogenic differentiation, while di-methylation of H3K4 as well as tri-methylation of H3K4, H3K36 and H3K27 show more stable profiles [11]. We could further demonstrate that only the cluster 1 genes with stable profiles are enriched for GO terms related to muscle development. Moreover, we overlapped the common cluster 1 genes of H3K4me2/3 from undifferentiated C2C12 cells with the common cluster 1 genes of H3K4me2/3 from the differentiated stage, which resulted in 267 genes with stable H3K4me2 and H3K4me3 profiles. Although methylation profiles are stable, we identified a considerable number of genes (58 out of 267 genes) differentially expressed upon differentiation of C2C12 cells, indicating an additional regulation by other factors. To further analyze the expression regulation of these common stable cluster 1 genes, we determined genome-wide binding of MyoD, which plays an essential role in activation of muscle-related genes [16,19,20,22,26,27].

MyoD typically binds promoters and enhancers of muscle-relevant genes to remodel the chromatin and activate transcription [22,26,49]. However, MyoD binding is not always associated with transcriptional activation [16]. For example, its repressive role was shown for the genes *Ccnb1* [23], *c-Fos* [24], and *Sp1* [25]. In our study, MyoD binding is significantly enriched in cluster 1 genes in undifferentiated and differentiated C2C12 cells. Interestingly, a subset of the differentially expressed genes with stable H3K4me2 and H3K4me3 profiles that are bound by MyoD in the myotubes are down-regulated, revealing a repressive potential of MyoD toward H3K2/3 marked genes. Five of the down-regulated genes harbor the preferred E-box motif in their MyoD peaks. The latter include Dusp6, which is a negative regulator of the MAP kinase superfamily and thus, plays a role in the regulation of proliferation and differentiation [54]. Moreover, Dusp6 expression is also negatively regulated by the MyoD cofactor Mef2a [55] in skeletal and cardiac muscle [56]. Another down-regulated gene is *Ptbp1*, an antagonist of RBM4, which in turn activates the selection of skeletal muscle-specific exons in alpha-tropomyosin mRNA [57]. Finally, the zinc finger TF Patz1 is an important regulator of pluripotency by maintaining embryonic stem cells in an undifferentiated state [52], suggesting that Patz1 plays a similar role in C2C12 cells. Moreover, Patz1 interacts with p53 to target genes that are associated with cell differentiation and apoptosis [58]. It is located in the DiGeorge syndrome region on chromosome 22q12 and plays a critical role in the control of cell growth and embryonic development, which has been demonstrated by neural tube and cardiac outflow tract defects in Patz1 knockout mice [59].

*Patz1* is ubiquitously expressed at early stages of embryonic development and becomes more restricted at later stages with almost no detectable expression in somites [59]. In contrast, *MyoD* shows an increased expression pattern in somites during embryonic development [60], indicating that *Patz1* may represent a target which can be negatively regulated by MyoD during skeletal muscle development. Indeed, we show that *Patz1* expression is strongly reduced, associated with MyoD binding at the *Patz1* promoter upon myogenic differentiation. However, it remains unclear how Patz1 regulates myogenic differentiation and further studies are needed to investigate the underlying mechanisms.

Out of the differentially expressed common stable cluster 1 genes, 40% (23 genes) show a differential MyoD binding, while 36% (21 genes) show a constitutive MyoD binding. The differential expression of the latter could possibly be explained by other cofactors necessary for transcriptional regulation. For example, several studies have shown that MyoD can activate gene transcription in cooperation with other factors such as E-proteins and the chromatin remodeling factor Baf60c [19–21].

In summary, we identified a subset of highly expressed genes related to muscle development, which show a stable H3K4me2 enrichment over the gene body and H3K4me3 enrichment towards the gene body during myogenic differentiation. Our study reveals a significantly higher binding of MyoD to this particular subset of genes that correlate with repression of transcription. Interestingly, further analysis and experiments revealed that MyoD binds and down-regulates *Patz1* during myogenic differentiation. This observation was further confirmed in MyoD driven differentiation of fibroblasts to muscle cells. These findings might provide an important regulatory mechanism to promote myogenic differentiation.

## Supporting information

S1 Table Overview of total and uniquely mapped reads per sample in ChIP-seq. Sequence reads (single-end 51 bp) were mapped to the mouse reference genome (mm9).
(XLSX)

S2 Table GO terms for H3K4me2 clusters in Undiff C2C12. Muscle-related GO terms are marked in red.
(XLSX)

S3 Table GO terms for H3K4me2 clusters in Diff C2C12. Muscle-related GO terms are marked in red.
(XLSX)

S4 Table GO terms for H3K4me2 cluster 1 genes in Undiff and Diiff C2C12. Muscle-related GO terms are marked in red.
(XLSX)

S5 Table GO terms for H3K4me3 clusters in Undiiff C2C12. Muscle-related GO terms are marked in red.
(XLSX)

S6 Table GO terms for H3K4me3 clusters in Diiff C2C12. Muscle-related GO terms are marked in red.
(XLSX)

S7 Table GO terms for H3K4me3 cluster 1 genes in Undiff and Diiff C2C12. Muscle-related GO terms are marked in red.
(XLSX)

S8 Table GO terms for H3K4me2/3 cluster 1 genes in Undiff and Diiff C2C12. Muscle-related GO terms are marked in red.
(XLSX)

S9 Table Number of genes in H3K4me2/3 clusters bound by MyoD. (XLSX)

S10 Table Repression of MYOD on target genes in IMR-90 cells cultured with growth medium (GM) and differentiation medium (DM). (XLSX)

S1 Fig Flow chart showing the filtering criteria and results for RefSeq genes (mm9). (TIFF)

S2 Fig Average profile of H3K4me2 and H3K4me3 in Undiff and Diff C2C12 cells. (A) Average profile of H3K4me2 and H3K4me3 in Undiff C2C12 and (B) Diff C2C12 cells around the transcription start site (TSS).
(TIFF)

S3 Fig Clustering analysis of H3K4me2 profiles in differentiated C2C12 cells. (A) H3K4me2 profiles identified by k-means clustering. The clustering is based on the transcription start site (TSS) and the corresponding number of genes is given for each cluster. Genes with multiple TSS can be present in more than one cluster. (B) The box plot (25% to 75% quartile) shows the levels of gene expression (FPKM values) of the different H3K4me2 clusters in Diff C2C12 cells. The expression of cluster 1 and cluster 2 genes was compared using the Mann-Whitney U test. (C) Overlap of genes between the clusters of H3K4me2 in Undiff and Diff C2C12 cells.
(TIFF)

S4 Fig Clustering analysis of H3K4me3 profiles in differentiated C2C12 cells. (A) H3K4me3 profiles identified by k-means clustering. The clustering is based on the transcription start site (TSS) and the corresponding number of genes is given for each cluster. Genes with multiple TSS can be present in more than one cluster. (B) The box plot (25% to 75% quartile) shows the levels of gene expression (FPKM values) of the different H3K4me3 clusters in Diff C2C12 cells. The expression of cluster 1 and cluster 2 genes was compared using the Mann-Whitney U test. (C) Overlap of genes between the clusters of H3K4me3 in Undiff and Diff C2C12 cells.
(TIFF)

S5 Fig Comparison of H3K4me2 and H3K4me3 cluster 1 in differentiated C2C12 cells. (A) Overlap of H3K4me2 and H3K4me3 cluster 1 genes in Diff C2C12 cells. The P value is based on a hypergeometric test. (B) GO enrichment analysis of common cluster 1 genes using the DAVID database. The top ten biological process terms with an adjusted (Benjamini-Hochberg) P value ≤ 0.01 are indicated. GO terms related to muscle development are highlighted in red.
(TIFF)

S6 Fig H3K4me2 and H3K4me3 profiles on selected genes. (A) The enrichment of H3K4me2 and H3K4me3 on selected muscle-relevant cluster 1 genes. The TSS is marked by an arrow. The y-axis indicates the ChIP-seq signal. (B) ChIP-qPCR validation of H3K4me2 and H3K4me3 occupancy on the selected genes.
(TIF)

S7 Fig Induction of MYOD in fibroblasts. (A) MYOD expression was measured by qPCR before and after induction in growth medium (GM) and differentiation medium (DM) IMR-90 fibroblasts. The expression of MYOD in control GM was set to 1. (B) Expression levels (mRNA) of PATZ1 in BJ fibroblasts. The expression of PATZ1 in control GM was set to 1. P value was calculated using Student's t-test based on at least three independent experiments.
(TIFF)
