# Supplementary figures and images for: Muscle-relevant genes marked by stable H3K4me2/3 profiles and enriched MyoD binding during myogenic differentiation
Source: PLoS One. 2017 Jun 13;12(6):e0179464. doi: 10.1371/journal.pone.0179464 (PMC5469484; doi:10.1371/journal.pone.0179464)

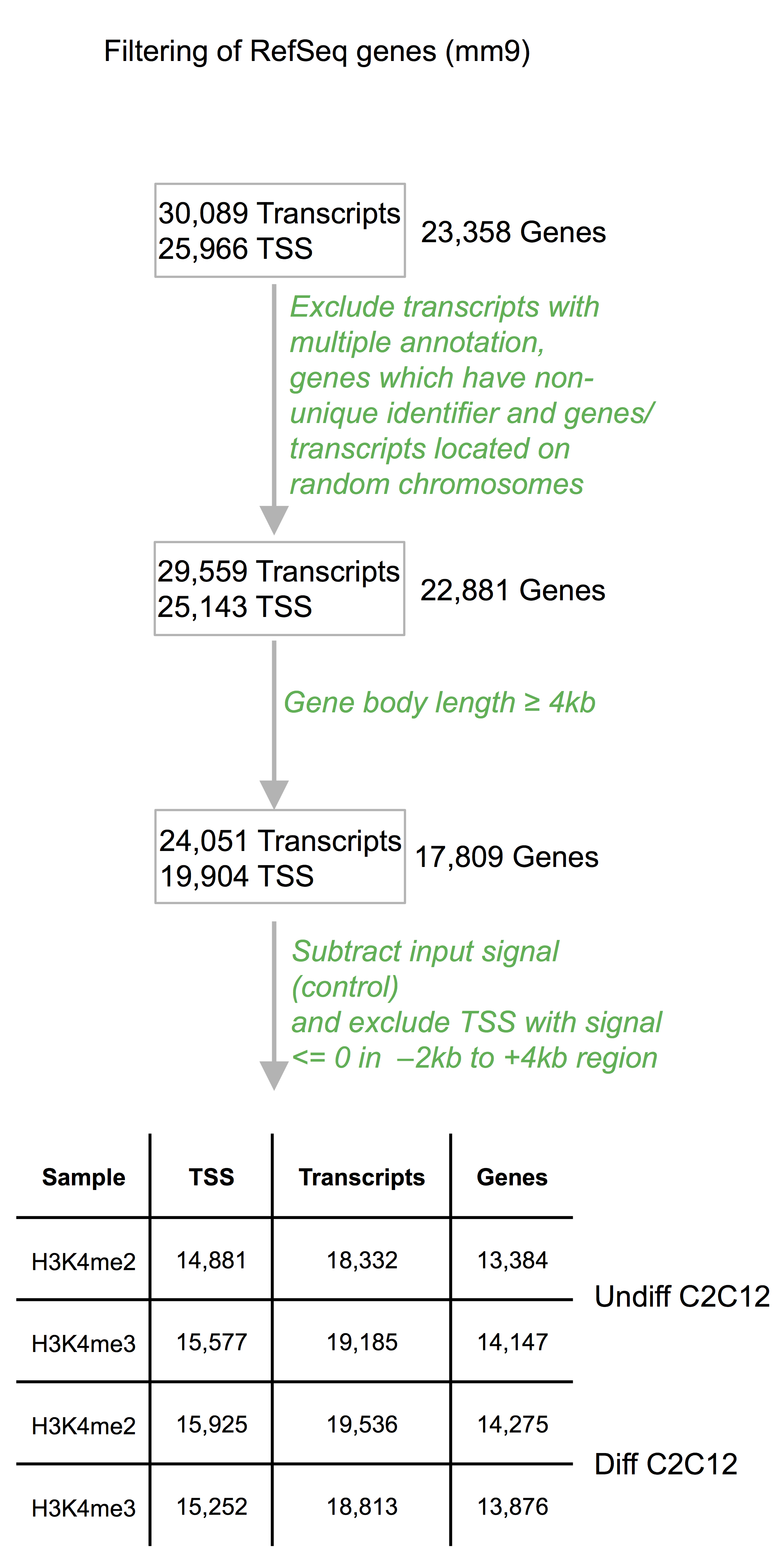

Supplement: S1 Fig — (TIFF) [file pone.0179464.s011.tiff]

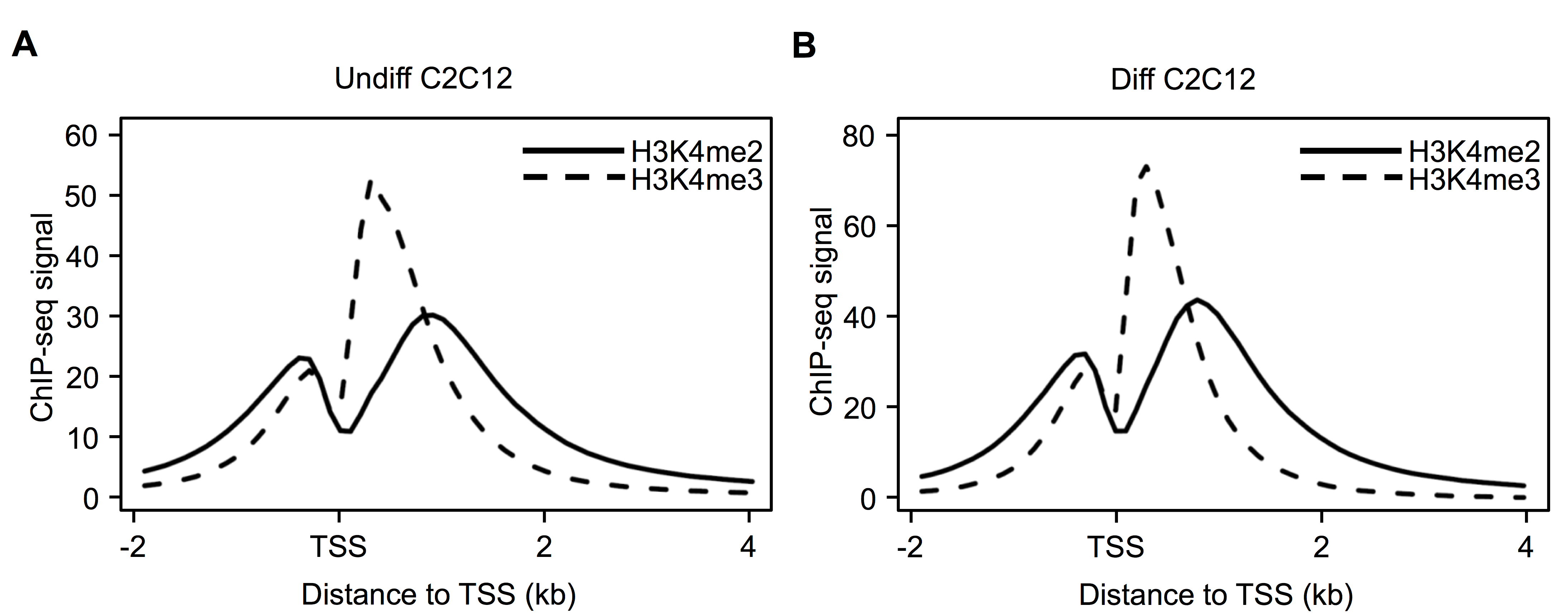

Supplement: S2 Fig — (A) Average profile of H3K4me2 and H3K4me3 in Undiff C2C12 and (B) Diff C2C12 cells around the transcription start site (TSS). (TIFF) [file pone.0179464.s012.tiff]

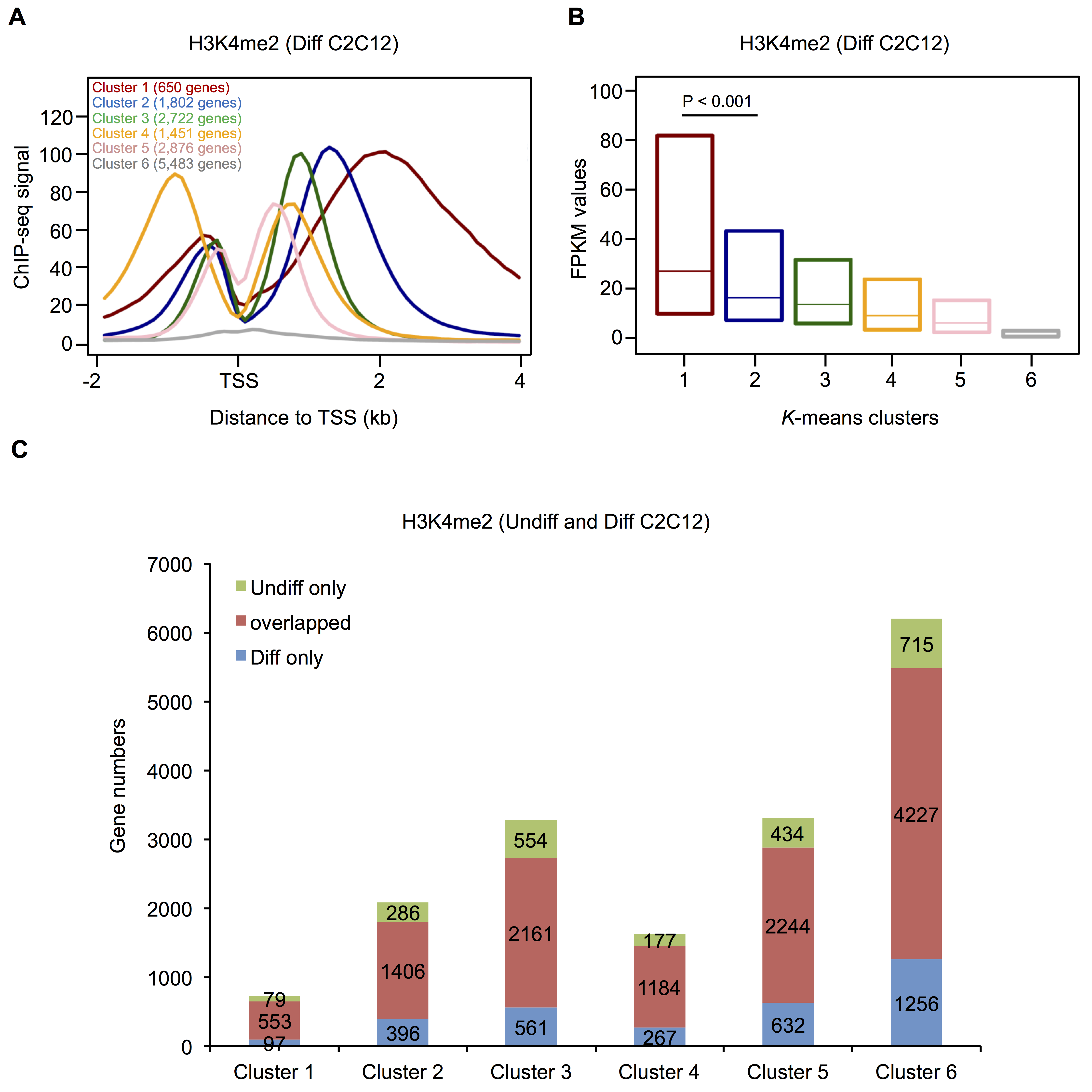

Supplement: S3 Fig — (A) H3K4me2 profiles identified by k-means clustering. The clustering is based on the transcription start site (TSS) and the corresponding number of genes is given for each cluster. Genes with multiple TSS can be present in more than one cluster. (B) The box plot (25% to 75% quartile) shows the levels of gene expression (FPKM values) of the different H3K4me2 clusters in Diff C2C12 cells. The expression of cluster 1 and cluster 2 genes was compared using the Mann-Whitney U test. (C) Overlap of genes between the clusters of H3K4me2 in Undiff and Diff C2C12 cells. (TIFF) [file pone.0179464.s013.tiff]

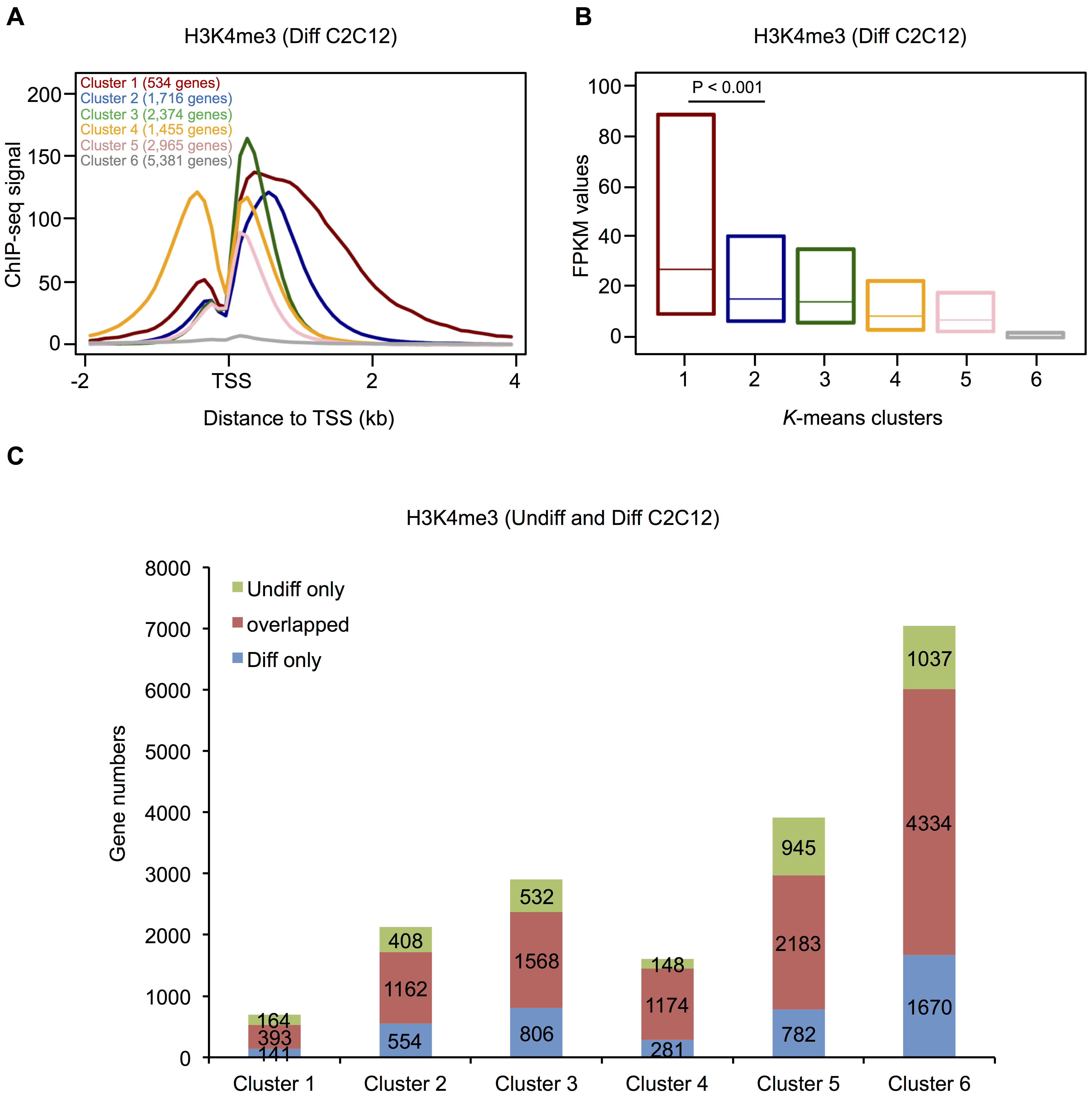

Supplement: S4 Fig — (A) H3K4me3 profiles identified by k-means clustering. The clustering is based on the transcription start site (TSS) and the corresponding number of genes is given for each cluster. Genes with multiple TSS can be present in more than one cluster. (B) The box plot (25% to 75% quartile) shows the levels of gene expression (FPKM values) of the different H3K4me3 clusters in Diff C2C12 cells. The expression of cluster 1 and cluster 2 genes was compared using the Mann-Whitney U test. (C) Overlap of genes between the clusters of H3K4me3 in Undiff and Diff C2C12 cells. (TIFF) [file pone.0179464.s014.tiff]

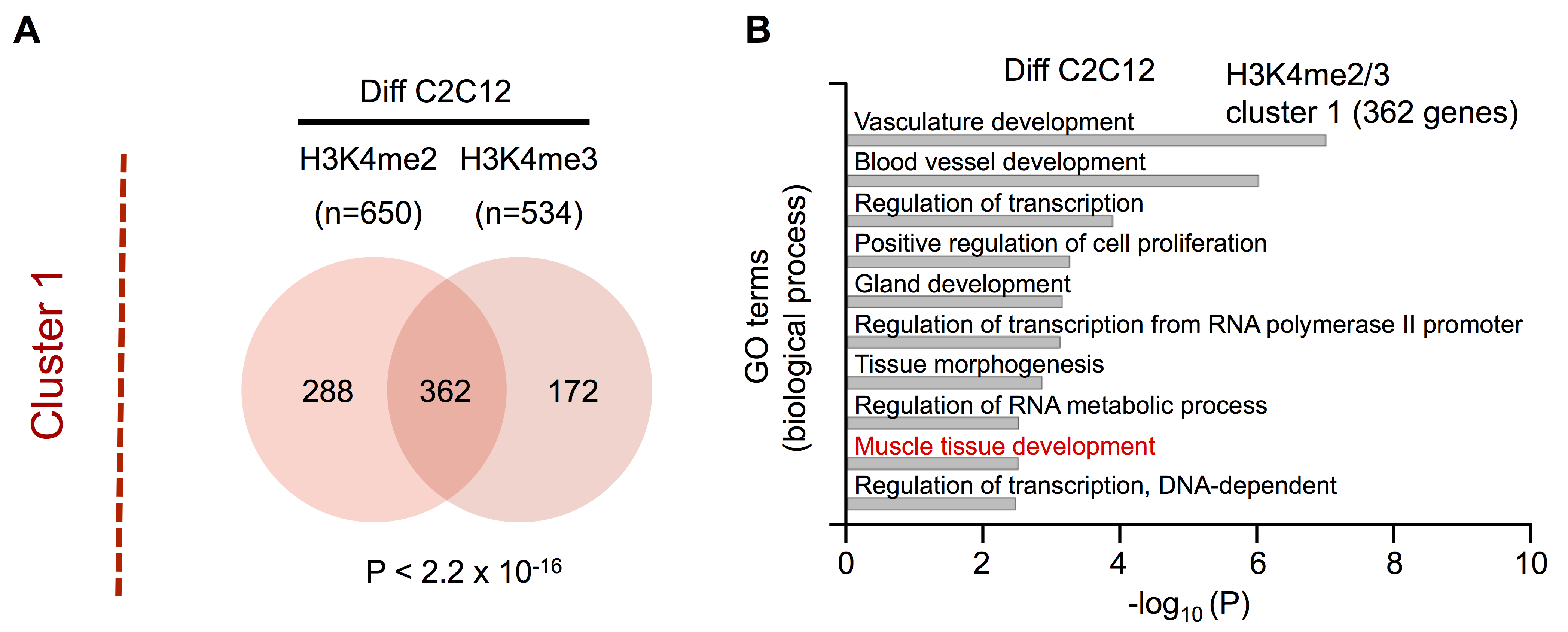

Supplement: S5 Fig — (A) Overlap of H3K4me2 and H3K4me3 cluster 1 genes in Diff C2C12 cells. The P value is based on a hypergeometric test. (B) GO enrichment analysis of common cluster 1 genes using the DAVID database. The top ten biological process terms with an adjusted (Benjamini-Hochberg) P value ≤ 0.01 are indicated. GO terms related to muscle development are highlighted in red. (TIFF) [file pone.0179464.s015.tiff]

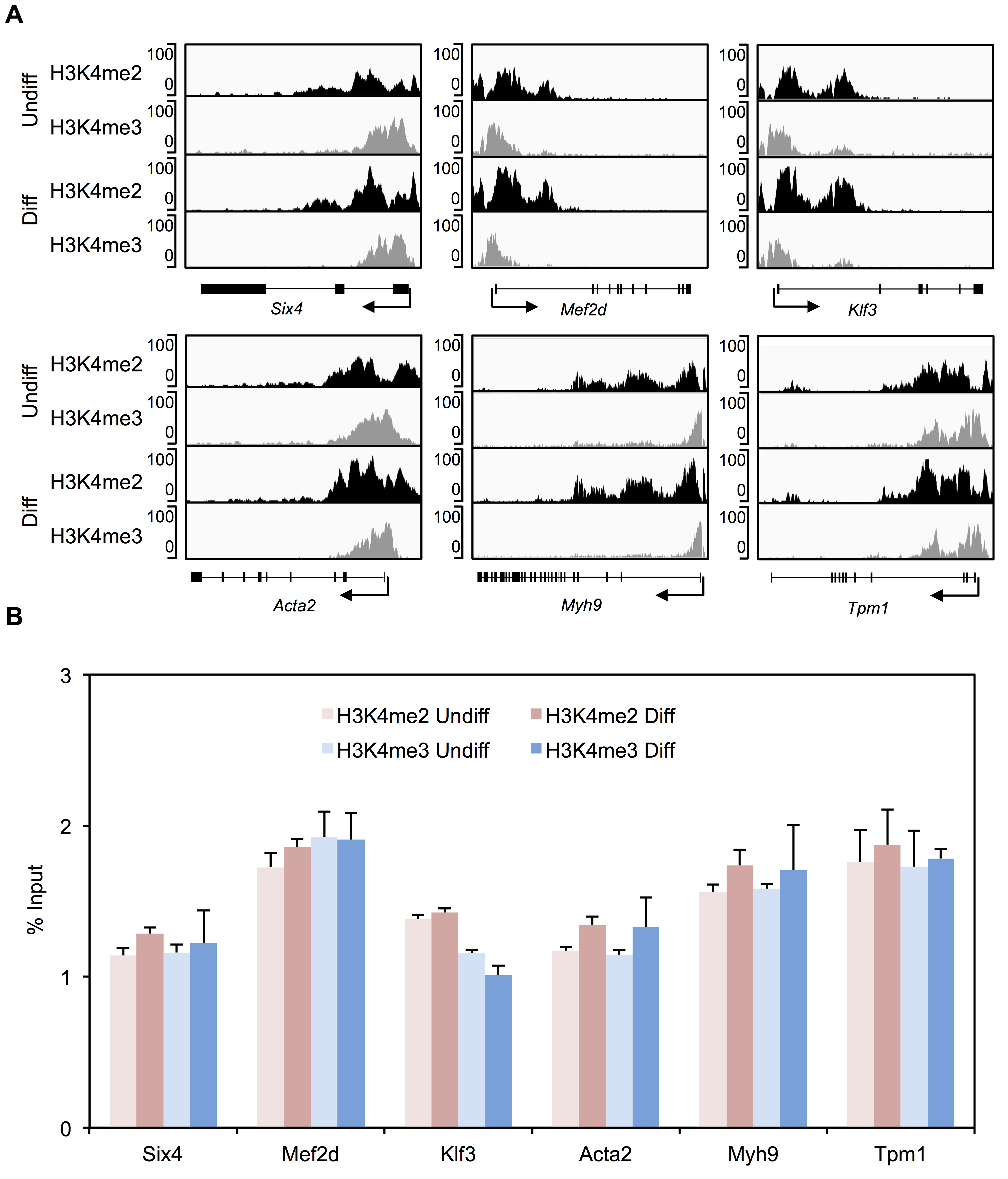

Supplement: S6 Fig — (A) The enrichment of H3K4me2 and H3K4me3 on selected muscle-relevant cluster 1 genes. The TSS is marked by an arrow. The y-axis indicates the ChIP-seq signal. (B) ChIP-qPCR validation of H3K4me2 and H3K4me3 occupancy on the selected genes. (TIF) [file pone.0179464.s016.tif]

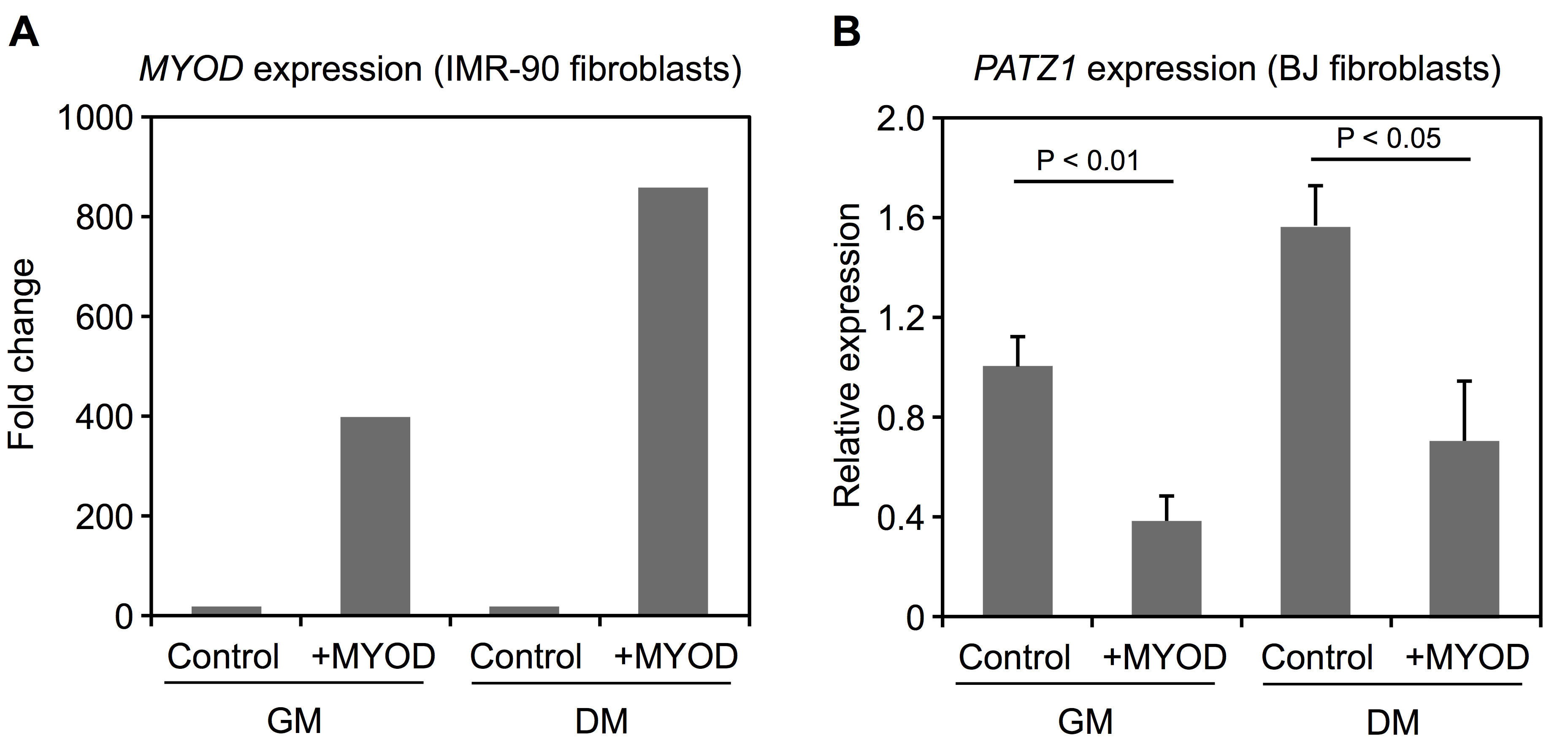

Supplement: S7 Fig — (A) MYOD expression was measured by qPCR before and after induction in growth medium (GM) and differentiation medium (DM) IMR-90 fibroblasts. The expression of MYOD in control GM was set to 1. (B) Expression levels (mRNA) of PATZ1 in BJ fibroblasts. The expression of PATZ1 in control GM was set to 1. P value was calculated using Student's t-test based on at least three independent experiments. (TIFF) [file pone.0179464.s017.tiff]
